# Off-axis multilayer zone plate with 16 nm × 28 nm focus for high-resolution X-ray beam induced current imaging

**DOI:** 10.1107/S1600577521006159

**Published:** 2021-07-22

**Authors:** Jakob Soltau, Lert Chayanun, Mikhail Lyubomirskiy, Jesper Wallentin, Markus Osterhoff

**Affiliations:** a Institute for X-ray Physics, University of Göttingen, Friedrich-Hund-Platz 1, 37077 Göttingen, Germany; b Synchrotron Radiation Research and NanoLund, Lund University, Box 118, 22100 Lund, Sweden; c Deutsches Elektronen-Synchrotron DESY, Notkestrasse 85, 22607 Hamburg, Germany

**Keywords:** X-ray beam induced current, XBIC mapping, multilayer zone plates, X-ray imaging, X-ray optic

## Abstract

Using multilayer zone plates (MZPs) as two-dimensional optics, focal spot sizes of less than 10 nm can be achieved, as we show here with a focus of 8.4 nm × 9.6 nm, but the need for order-sorting apertures prohibits practical working distances. To overcome this issue, here an off-axis illumination of a circular MZP is introduced to trade off between working distance and focal spot size. By this, the working distance between order-sorting aperture and sample can be more than doubled. Exploiting a 2D focus of 16 nm × 28 nm, real-space 2D mapping of local electric fields and charge carrier recombination using X-ray beam induced current in a single InP nanowire is demonstrated. Simulations show that a dedicated off-axis MZP can reach sub-10 nm focusing combined with reasonable working distances and low background, which could be used for *in operando* imaging of composition, carrier collection and strain in nanostructured devices.

## Abstract 2

In an off-axis geometry, a circular multilayer zone disc is used to locally generate X-ray beam induced currents inside a single InP nanowire. This allows to spatially probe local electric fields at practical working distances and very low background signal.

## 1. Introduction   

For hard X-ray imaging of bulk materials and biological specimens, the development of diffractive optics such as zone plates or multilayer lenses has been transformative in recent years (Döring *et al.*, 2013 ▸; Mohacsi *et al.*, 2017 ▸; Bajt *et al.*, 2018 ▸). Samples can now be probed *in situ* or *in vivo* with nanometre resolution (Victor *et al.*, 2018 ▸). In optics with large numerical apertures (NAs), used to obtain the smallest focal spot sizes, the diffractive elements need to be scaled down since the size of the smallest structure corresponds directly to the size of the focal spot. The most common diffractive optics for focusing X-rays are Fresnel zone plates (FZPs) which are fabricated using lithography. FZPs were developed for the focusing of soft X-rays (Niemann *et al.*, 1974 ▸; Schneider, 1998 ▸; Gorelick *et al.*, 2019 ▸; Rösner *et al.*, 2020 ▸) where zone widths of down to 8 nm were achieved. But for hard X-rays lithographic fabrication methods are limited to approximately 20 nm smallest structure sizes (Chang & Sakdinawat, 2014 ▸). A smaller focal spot size can be achieved by using double-sided FZPs, for X-ray wavelengths of several nanometre and down to 1 Å (Mohacsi *et al.*, 2017 ▸). In contrast, multilayer zone plates (MZPs) are diffractive optics with smallest structure elements of down to 5 nm (Eberl *et al.*, 2014 ▸), achieved using pulsed laser deposition (PLD). However, the focal length of currently 1 mm at 15 keV results in short working distances. On the other hand, the fabrication allows the usage of MZPs at energies up to 100 keV (Osterhoff *et al.*, 2017*b* ▸).

**Figure 1:**
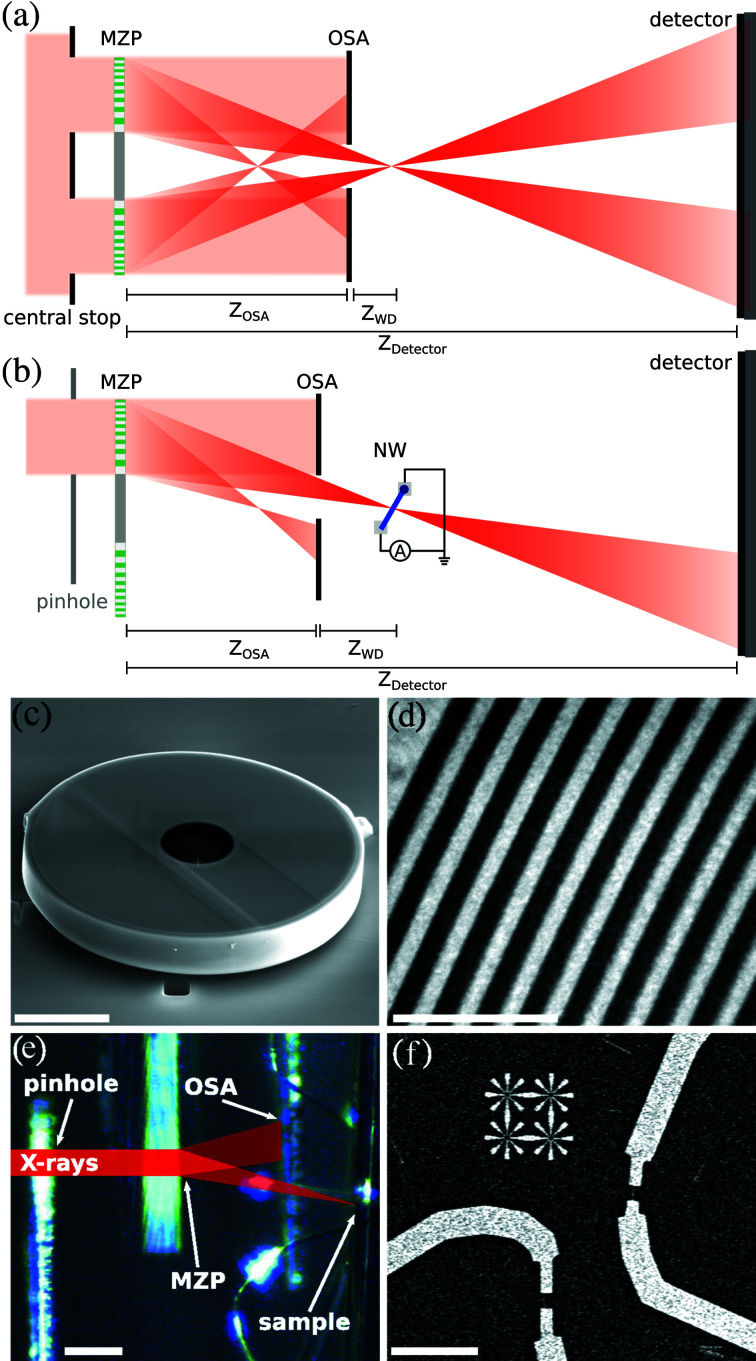
Two setups using an MZP in combination with apertures. Both are illuminated by an X-ray beam pre-focused using CRLs. (*a*) Classic setup with a fully illuminated MZP and a central stop combined with an OSA to block background photons. The OSA is positioned to block the second-order focus. (*b*) Setup with an off-axis illuminated MZP. Note that the pinhole can be omitted if the incoming beam is already confined by optics upstream. The contacted nanowire (NW) is depicted at the focal plane. The OSA is positioned to block the second-order focus. (*c*) Scanning electron microscopy (SEM) image of the MZP mounted on a Si_3_N_4_ window. (*d*) SEM image of the outer zones of the MZP, showing a width of down to 5 nm. (*e*) Photograph (side-view) of the off-axis setup at the P10 beamline. The pinhole and the OSA are visible, the Si_3_N_4_ window where the MZP is mounted (not visible) and the nanowire sample with the bond wires. (*f*) SEM of the nanowire devices and the star-shaped test structures, both created using lithography. (*a*,*b*) Not to scale. Scalebars: (*c*) 5 µm, (*d*) 50 nm, (*e*) 250 µm, (*f*) 100 µm.

Besides a small focus size, a low background signal is of importance in most experiments such as X-ray fluorescence (XRF) (Yan *et al.*, 2016 ▸; Deng *et al.*, 2017 ▸), ptychography (Holler *et al.*, 2014 ▸; Shi *et al.*, 2019 ▸), scanning SAXS or WAXS (Riekel *et al.*, 2019 ▸), for new imaging schemes such as Compton X-ray microscopy (Villanueva-Perez *et al.*, 2018 ▸) or, as shown in this manuscript, for X-ray beam induced current (XBIC) (Buonassisi *et al.*, 2003 ▸). Unfortunately, when using diffractive X-ray optics, photons are diffracted into several orders (negative or positive) and some photons are undiffracted (zeroth order), resulting in a background signal and affecting the sensitivity and contrast of many measurements. The standard procedure to prevent this is blocking ‘unwanted’ photons by a pair of apertures. Therefore a first central stop is positioned in front of the optics, and a pinhole is used as an order-sorting aperture (OSA) positioned between the optics and the sample in the vicinity of the focal spot [see Fig. 1 ▸(*a*)]. The working distance *z*_WD_ is the distance between OSA and focal spot. It depends on the radius of the central stop. A small *z*_WD_ imposes strong restrictions on the study of samples. Due to volume diffraction effects in multilayer optics, also even diffraction orders occur (Maser *et al.*, 2004 ▸), resulting in the formation of a second-order focus at the position of half the focallength, reducing *z*_WD_ even further. To increase *z*_WD_, the radius of the central stop needs to be enlarged and thereby more inner zones of the optic are not illuminated. This results in a loss of photon flux. However, the maximum length of *z*_WD_ is generally limited to half the focal length by the position of the second-order focus.

An alternative approach to a large central stop for filtering the beam from photons of unwanted diffraction orders is to separate the focused X-rays from the optical axis of the incoming beam, which could be advantageous for three important reasons: (i) The central part of the beam no longer needs to be blocked and therefore the central stop can be omitted or replaced by a simple pinhole. This results in an increased efficiency since the highest photon flux is in the center of the beam. (ii) Besides a reduced efficiency, the height of the focus side maxima is dependent on the ratio of the blocked area relative to the illuminated area (Simpson & Michette, 1984 ▸). This is not the case for an off-axis illumination, as shown in the supporting information using finite-difference simulations. The simulations show an increased focus side maxima intensity by a factor of 2.3 for the case of a fully illuminated MZP and an off-axis MZP with the same NA. Strong focus side maxima are reducing the effective resolution. (iii) In the case of off-axis illumination, the OSA can be placed further upstream [see Fig. 1 ▸(*b*)], even beyond the second-order focus, without the limitations (i) and (ii), leaving more space for the sample. The compromise when it is placed further upstream is the reduced NA, thus the ratio of the outermost zone width to the possible focus size is no longer given. Nevertheless, this off-axis configuration is used in most experiments using a pair of one-dimensional optics such as multilayer Laue lenses (MLLs) (Morgan *et al.*, 2015 ▸). The reduced dimensions of one-dimensional optics imposes the disadvantage that, for generating a 2D focus, two optics are required. Since the two MLLs need to be aligned in close distance, the complexity of the setup is further increased. In addition, the MLLs must be aligned such that both focal planes match. When the photon energy changes, readjustment becomes necessary, which limits the flexibility in an experiment.

In this manuscript, we show that for high-resolution X-ray microscopy this off-axis configuration is not limited to a pair of 1D lenses but is also possible for a single circular diffractive optical element. We compare the off-axis geometry with a classical zone plate geometry and evaluate them using ptychography and finite-difference simulations. In Section 3.2 we show that using an off-axis illuminated MZP it is possible to focus X-rays to a focal point separated from the beam. We measure focus sizes of 16.2 nm × 27.9 nm and 8.4 nm × 9.6 nm for the off-axis and the classic geometries, respectively (see Section 3.1).

Crucially, the off-axis geometry more than doubles the distance between the OSA and the focus, increasing the working distance from 0.18 mm to 0.44 mm. We take advantage of the improved working distance and reduced background to perform simultaneous high-resolution XBIC real-space mapping and holographic, ptychographic or scanning transmission imaging of semiconductor nanowires, as shown in Section 3.3. Finally, we use the nanowire as a detector to measure the intensity distributions along the optical axis of an off-axis illuminated MZP in Section 3.4.

## 2. Methods   

### 2.1. The multilayer zone plate   

The MZP was fabricated using the technique of pulsed laser deposition (PLD), following the Fresnel zone plate formula with an outermost layer thicknesses of 5 nm, a diameter of 15.6 µm, 784 zones and a thickness of 2.4 µm [see Figs. 1 ▸(*c*) and 1(*d*)]. The Fresnel zone plate formula defines the radius of a zone as *r* ≃ (*n*λ*f*)^1/2^ with λ the wavelength, *f* the focal length and *n* the index of the zone. The outermost zone width Δ*r*_*N*_ defines the maximum diffraction angle of the first order, which limits the NA of the optics and the size of the focal spot as a result. Therefore Δ*r*_*N*_ is almost equivalent to the theoretical focal spot size. MZPs have been used for focusing X-rays in an energy range from 8 keV (Döring *et al.*, 2013 ▸) up to 100 keV (Osterhoff *et al.*, 2017*b* ▸).

A round glass wire of diameter 2.1 µm was used as a substrate for the deposition process. Within our parameters, the glass wire corresponds to the first 13 zones, which therefore are missing. By using tapered fibers with an opening angle of 2.5 mrad the zones become tilted relative to the beam axis and in the direction of the focus. This enhances the X-ray focusing efficiency (Yan *et al.*, 2010 ▸). In the case of the MZP used here, simulations show that this increases the focusing efficiency by almost a factor of three (see supporting information). In the fabrication process, the slice position is determined on the basis of the outer radius of the MZP. As materials for the zones, Ta_2_O_5_ and ZrO_2_ were used. One advantage of PLD over other sputtering techniques originates in the energetic bombardment, resulting in cumulative smoothing (Röder *et al.*, 2010 ▸) which decreases roughness and distortions (Eberl *et al.*, 2014 ▸).

The mounting of the MZP was modified compared with previous experiments, to achieve a better long-term stability during the measurements and to simplify the alignment process. In previous experiments the MZP was mounted on a tungsten tip, but here the MZP was mounted flat with three contacts on a Si_3_N_4_ window (thickness 1 µm) using a focused ion beam (FIB) setup [see Fig. 1 ▸(*c*)]. The Si_3_N_4_ window enables the precise pre-alignment of the MZP using the reflection on the window by a laser beam which is aligned parallel to the X-ray beam. Further, the flat mounting on the Si_3_N_4_ window fixed by three contacts increases the stability against potential oxidation processes at the contacts.

### 2.2. X-ray beam induced current (XBIC)   

XBIC can be used to measure local electric fields and charge carrier recombination conditions. Compared with scanning photocurrent microscopy (SPCM) (Ahn *et al.*, 2007 ▸), a smaller diffraction limit allows for higher spatial resolution (Chayanun *et al.*, 2019*a* ▸); compared with electron beam induced current (EBIC) (Hanoka, 1980 ▸), a higher penetration depth allows to characterize thicker samples (Stuckelberger *et al.*, 2015 ▸). Thus, complete devices can be investigated *in operando*.

In XBIC, an absorbed X-ray photon excites secondary electrons in semiconductors through a cascade process, which then thermalize to the band edge of the semiconductor (Rodnyi, 1997 ▸; Gektin & Korzhik, 2017 ▸). These secondary charges are collected under an applied or built-in electric field within the measured device. The XBIC signal is therefore dependent on the local electric field and carrier recombination conditions, and by scanning the sample in an X-ray focus the XBIC technique can be used to map the local charge transport and charge collection properties of semiconductor devices. For the XBIC measurements at the GINIX beamline, see below, the XBIC measurement system was integrated into the control system at the instrument, as described previously (Chayanun *et al.*, 2019*a* ▸). Nanowire devices were fabricated on an Si_3_N_4_ window, wire bonded and mounted in a special sample holder with electrical connections. Test structures, similar to Siemens stars, were also deposited on the Si_3_N_4_ window next to the nanowire device. The structure was used for a characterization of the off-axis beam path by ptychography. The smallest features of the star-shaped test pattern were 100 nm.

### 2.3. P10-GINIX setup   

The off-axis configuration of the MZP and XBIC measurements were performed at the GINIX instrument (Kalbfleisch *et al.*, 2011 ▸) at the coherence beamline P10, at the PETRA III storage ring (Hamburg, Germany). The experimental setup is depicted in Fig. 1 ▸(*b*). The undulator beam at the P10 beamline was monochromated [Si(111) channel-cut monochromator] to a photon energy of 13.8 keV and pre-focused by a compound refractive lens (CRL). The MZP, apertures and nanowire device were mounted on the high-resolution stage of the instrument (Osterhoff *et al.*, 2017*a* ▸) for fly scans and reduced vibrations, especially with respect to each other. As depicted in Fig. 1 ▸(*b*), the pinhole was mounted in front of the MZP and the OSA was carefully aligned between the MZP and the sample. The diameters of the apertures were 5.6 µm for the pinhole and 3.5 µm for the OSA. The focal length of the MZP at 13.8 keV is 0.92 mm. The maximum distance between the OSA and the focus is 0.44 mm. A photograph of the setup is shown in Fig. 1 ▸(*e*). The pinhole was installed to decouple from vibrations of the incoming beam. The diffraction patterns of the X-ray beam were recorded using a single-photon-counting pixel detector (Eiger 4M, Dectris Ltd, Switzerland) positioned at *z*_DE_ = 5.1 m. The detector has 2162 × 2068 pixels of size Δ_px_ = 75 µm.

### 2.4. P06-Nanoprobe setup   

In addition to the measurement at the GINIX setup using an off-axis illuminated MZP, a second measurement was performed to determine the focus size of a fully illuminated MZP as a reference. Since the OSA had to be aligned at close distance to a test structure for this purpose, the measurement was realized in a separate experiment. This experiment was realized using the PtyNAMI instrument (Schropp *et al.*, 2020 ▸) at the Hard X-ray Micro/Nano-Probe at the beamline P06, which is also positioned at the PETRA III storage ring [see Schroer *et al.* (2016 ▸) for details]. The MZP had the same zone parameters as the one used at the GINIX setup. The undulator beam was monochromated [Si(111) channel-cut monochromator] to a photon energy of 15 keV, and then pre-focused using a CRL optic with the MZP positioned in its focal plane. The size of the CRL focus was larger than the diameter of the MZP to prevent a decrease in intensity at the outer MZP-zones.

In Fig. 1 ▸(*a*) the experimental setup of the fully illuminated MZP is depicted. Despite the two setups being quite similar, they differ in two important points:

(1) The shape of the first aperture. The aperture in this configuration is a solid central stop of 6 µm diameter for blocking the photons.

(2) The smallest possible distance between the OSA and the focus in the full-illumination configuration is 0.18 mm, less than half the distance as in the off-axis geometry. At a greater distance between the OSA and the focal plane the OSA would cut into the beam. The focal length of the MZP at 15 keV is slightly larger with a length of 1.0 mm. As a sample a Siemens star test structure with 50 nm smallest feature size was positioned at a distance of 1.07 mm relative to the MZP and 0.25 mm relative to OSA. The diffraction patterns of the X-ray beam were recorded using a single-photon-counting pixel detector (Pilatus 300k, Dectris Ltd, Switzerland) positioned at a distance relative to the optic of *z*_DE_ = 3.43 m. The detector has 619 × 487 pixels and a pixel size of Δ_px_ = 172 µm. Basic parameters of the two beamline endstations are given in Table 1 ▸.

## 3. Results   

### 3.1. Beam characterization with fully illuminated MZP   

The focus size and beam path of the MZP were characterized using ptychography (Rodenburg *et al.*, 2007 ▸). As mentioned, the experiment using the fully illuminated MZP was performed at the P06 nanoprobe instrument. The sample (Siemens star) was scanned with 25 × 51 scan points at 0.2 s acquisition time per frame. The reconstruction was performed using the ptychography code of the beamline based on the ePIE algorithm (Maiden & Rodenburg, 2009 ▸). The pixel size in the object plane was 3.2 nm.

**Figure 2:**
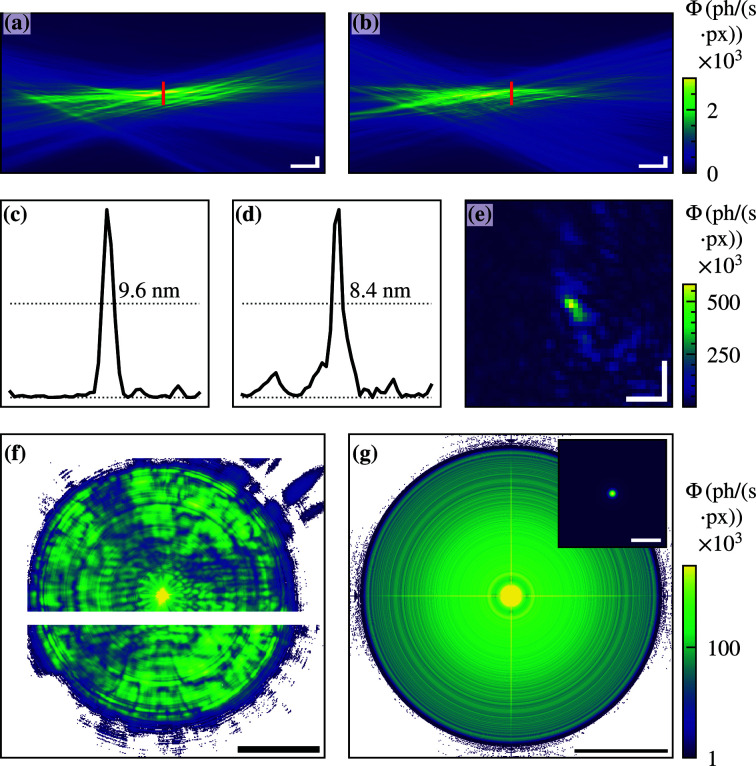
Ptychographic reconstruction of the fully illuminated MZP. The pixel size was 3.2 nm. (*a*,*b*) Back-propagated wavefields. (*c*,*d*) Line profiles showing a FWHM of 8.4 nm × 9.6 nm. (*e*) Intensity distribution in the focal plane. (*f*) Measured mean far-field intensity. The white bar is due to a detector gap. (*g*) Far-field intensity distribution obtained by FD simulations. The inset shows the simulated focal spot with a FWHM of 5.9 nm × 5.9 nm. The color bar of (*f*,*g*) is scaled logarithmically. Scalebars: (*a*,*b*) vertical 50 nm, horizontal 10 µm, (*c*–*e*) 25 nm, (*f*,*g*) *q* = 0.26 nm^−1^, inset (*g*) 25 nm.

Figures 2 ▸(*a*) and 2(*b*) show the reconstructed and back-propagated probe in the vicinity of the focus. The corresponding reconstructed object and probe can be found in the supporting information. The focus is shown in Figs. 2 ▸(*c*)–2(*e*) and has a FWHM of 8.4 nm × 9.6 nm. The FWHM was computed using the Python scipy.signal.peak_widths (Virtanen *et al.*, 2020 ▸). The corresponding mean recorded detector field is shown in Fig. 2 ▸(*f*), showing the highly divergent beam. The focusing efficiency of the fully illuminated MZP was determined to be 7.3%.

Note that the photon flux density of the back-propagated probe is not evenly distributed, since the limited commissioning beam time did not allow for better alignment of the MZP. Nevertheless, the distance between the OSA and the focus is fairly short (<180 µm), preventing the alignment of the nanowire device for XBIC measurements due to bond wires protruding from the substrate.

The measurements were compared with simulations, which were performed using a finite differences (FD) solver (Melchior & Salditt, 2017 ▸) in three dimensions. Dynamical diffraction effects such as multiple diffractions and volume effects were considered. The parameters of the simulated MZP were equivalent to the specifications of the MZP used in the XBIC experiment. The far-field pattern shows the strongly divergent beam [see Fig. 2 ▸(*g*)] and is similar to the far-field pattern measured in the experiment [see Fig.2 ▸(*f*)]. While the diffracted photons are distributed over the full detector, the non-diffracted photons are detected in only one pixel, which in a real detector pixel would result in beam damage, showing the necessity of apertures or beamstops. A characterization of the simulated focal spot gives a FWHM of 5.9 nm × 5.9 nm [see inset Fig. 2 ▸(*g*)]. Thus, the simulations give a similar focus size as the outermost zone width, and agree reasonably well with the measurements. The difference can be attributed to the slight misalignment already mentioned, as well as to possible local manufacturing deviations from the ideal shape.

### 3.2. Beam characterization with off-axis illuminated MZP   

**Figure 3:**
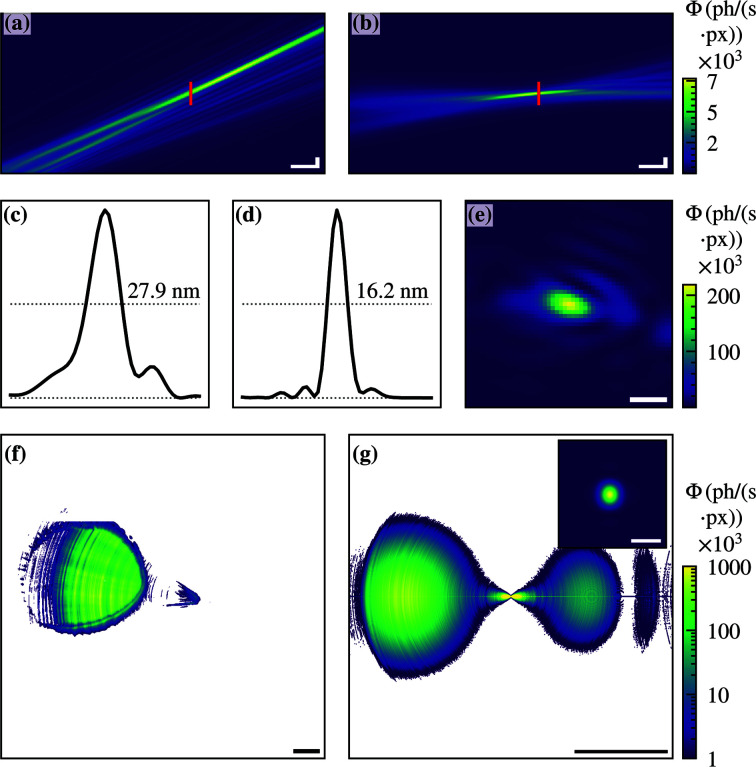
Ptychographic reconstruction of the off-axis illuminated MZP. The pixel size was 3.0 nm. (*a*, *b*) Back-propagated wavefield. (*c*, *d*) Line profiles from the same plane showing a FWHM of 16.2 nm × 27.9 nm. (*e*) Intensity distribution in the focal plane. (*f*) Measured mean far-field intensity distribution. (*g*) Far-field intensity distribution obtained by FD simulations without apertures. On the left side the focused beam of the positive first order is seen, while on the right side the divergent beam of the negative first order. The inset shows the simulated focal spot with a FWHM of 12.1 nm × 15.4 nm. The color bar of (*f*,*g*) is scaled logarithmically. Scalebars (same as Fig. 2 ▸): (*a*,*b*) vertical 50 nm, horizontal 10 µm, (*c*–*e*) 25 nm, (*f*) *q* = 0.10 nm^−1^, (*g*) *q* = 0.26 nm^−1^, inset (*g*) 25 nm.

The beam characterization of the off-axis illuminated MZP was performed at the GINIX endstation at the P10 beamline. The scan for ptychography reconstruction was done with 41 × 41 scan points and a 1.0 s acquisition time per frame. For reconstruction our own ptychography code based on the ePIE (Maiden & Rodenburg, 2009 ▸) algorithm was used. The pixel size in the object plane was 3.0 nm. The star-shaped test structure on the Si_3_N_4_ window of the nanowire device was used as a sample. Figures 3 ▸(*a*) and 3(*b*) show the reconstructed and back-propagated probe. Whereas the off-axis illumination in the vertical direction is only marginal [see Fig. 3 ▸(*b*)], the horizontal angle of the beam relative to the initial beam axis is clearly visible [see Fig. 3 ▸(*a*)].

The corresponding reconstructed object and probe can be found in the supporting information. The focal spot has a FWHM of 16.2 nm × 27.9 nm [see Figs. 3 ▸(*c*)–3(*e*)]. The reconstruction reveals an astigmatism based on a small mis­alignment of the focused X-ray beam. The difference in the focal plane positions in the vertical and horizontal directions can be estimated from Figs. 3 ▸(*a*) and 3(*b*) to be ∼16 µm. The corresponding mean detector image is shown in Fig. 3 ▸(*f*). The focused and thereby enlarged beam can be seen on the detector (left side) separated from some residual photons on the initial beam axis (center). The focusing efficiency of the off-axis illuminated MZP was determined to be 8.4%.

Equivalent to the fully illuminated MZP, FD simulations were performed for the off-axis illuminated MZP. In Fig. 3 ▸(*g*) the simulated far-field diffraction pattern is shown. In the left part of the far-field area the diffraction pattern of the positive first order can be seen, which generates the focal spot. In the right part the negative first order is visible and in between the non-diffracted photons of the zeroth diffraction order. The latter two diffraction orders justify the need for the OSA, and are therefore not visible in the measured detector image in Fig. 3 ▸(*f*). In contrast to the fully illuminated MZP, the pattern of the negative and positive diffraction orders do not overlap and can be distinguished.

The simulated focal spot has a width of 12.1 nm × 15.4 nm. The difference compared with the measured data can be explained by the astigmatism of the beam. Nevertheless, by using the off-axis illumination of the MZP, the distance between the OSA and the sample was more than doubled, enabling alignment of the nanowire device in the focal plane.

Before analysing the XBIC measurements, other off-axis X-ray optics will be addressed for comparison. Kirkpatrick–Baez (KB) mirrors are the most common off-axis optic for hard X-rays which focus the incoming beam by total reflection and obtain thereby high focusing efficiencies. Since their surface roughness is quite sensitive, KB mirrors are usually stored in vacuum tanks, which are permanently installed. To compensate for side maxima of the focus, KB mirrors are often used in combination with apertures. Unlike diffractive optics, KB mirrors cannot be used for direct imaging and are therefore only used for probing the sample. The focus size of KB mirrors is limited by the critical angle of reflection which can only be overcome by coating the mirrors with a multilayer structure. Using a coated KB mirror a focus of 12 nm × 13 nm at an X-ray energy of 33.6 keV was achieved (Da Silva *et al.*, 2017 ▸). But, the efficiency of diffractive multilayer optics can be further improved by using a wedged geometry, where all layers are tilted according to Bragg’s law. As a result, the focusing efficiency is no longer limited mainly by the diffraction efficiency itself, but only by the absorption within the multilayer optics (Yan *et al.*, 2007 ▸). First characterizations of wedged multilayer optics in one dimension have been performed, resulting in an efficiency of 69% (Bajt *et al.*, 2018 ▸). For off-axis MZPs manufactured in the future, the already tilted geometry can be changed to a wedged geometry, to further increase the focusing efficiency.

### 3.3. Characterization of the nanowire device   

**Figure 4:**
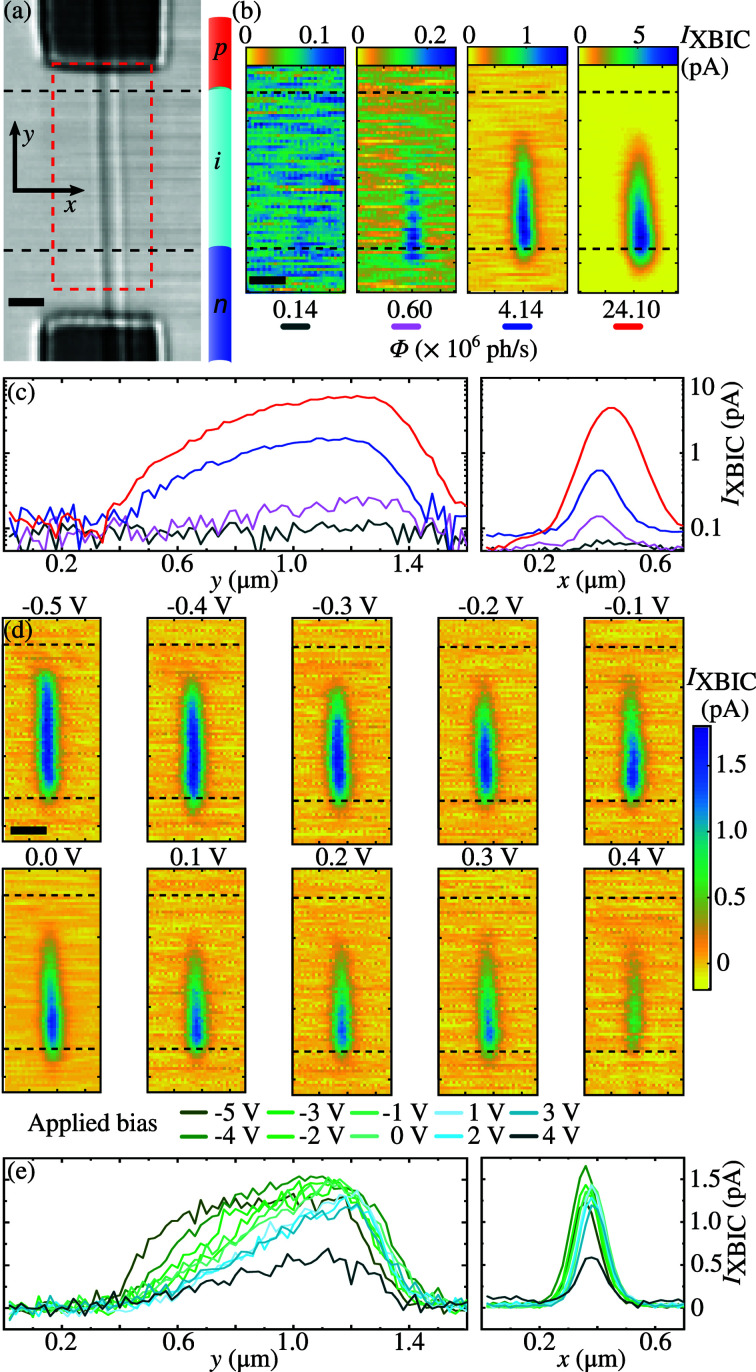
High-resolution XBIC maps using the off-axis illuminated MZP with a step size of 20 nm. (*a*) STXM image of a single nanowire device. Next to the STXM image is a schematic of a nanowire roughly indicating the doping segments. (*b*) XBIC maps from the sketched red square in (*a*), using different X-ray fluxes of a transmission of 0.6%, 2.5%, 17.2% and 100% of the maximum X-ray photon flux (Φ = 2.41 × 10^7^ photons s^−1^). Scalebars: 250 nm. The horizontally dashed lines indicate the doping junctions. (*c*) Axial and radial XBIC profiles, in logarithmic scale. (*d*) XBIC maps at different applied biases using Φ = 4.14 × 10^6^ photons s^−1^. (*e*) Axial and radial XBIC profiles, extracted from the XBIC maps in (*d*). Scalebars: 250 nm.

XBIC has been used to investigate the nanoscale carrier dynamics in many types of semiconductors, using established X-ray focusing methods (Zapf *et al.*, 2020 ▸; Chayanun *et al.*, 2019*b* ▸; Stuckelberger *et al.*, 2017 ▸). The spatial resolution is limited by the X-ray focus size, reaching around or slightly below 50 nm with established optics, and it is highly desirable to base XBIC on novel high-resolution optics such as MZPs. Therefore, the off-axis illuminated MZP was used to investigate single contacted p-i-n doped InP nanowire devices using XBIC at the P10-GINIX instrument. The nanowires were similar to the devices in our previous publication (Chayanun *et al.*, 2019*b* ▸), and were synthesized at Lund NanoLab, Lund University, Sweden, for advanced solar cells (Otnes *et al.*, 2018 ▸). We used the above-mentioned investigations to validate the present XBIC measurements using the new off-axis MZP configuration. The nanowire diameter is 180 nm, with a length of ∼3.3 µm. The nanowires themselves consist of three differently doped segments (p,i,n) which have a length of about 1.1 µm, see Fig. 4 ▸(*a*). Nanowires were transferred from the growth substrate onto a pre-defined Si_3_N_4_ membrane substrate. Then, they were turned into a single contacted nanowire device using electron beam lithography and metal evaporation (Chayanun *et al.*, 2019*b* ▸).

The geometry and sample we used allowed simultaneous collection of scanning transmission X-ray microscopy (STXM) and XBIC maps. Figure 4 ▸(*a*) shows a STXM image and Fig. 4 ▸(*b*) shows the corresponding 2D-XBIC maps at different photon fluxes (step size 20 nm, 0.1 s per point, fly scan). The two contacts and the nanowire are resolved, and the sketch and the dashed lines indicate the segment junctions. Most of the XBIC signal (*I*_XBIC_) was detected within the intrinsic middle segment of the nanowire [see Fig. 4 ▸(*b*)], where there is a built-in electric field as a result of a depletion region. The X-ray flux variation measurement was performed at zero bias (0 V). The transmission, *T*, of 0.6%, 2.5%, 17.2% and 100% of the maximum X-ray photon flux (Φ = 2.41 × 10^7^ photons s^−1^) was changed by using attenuation filters. Φ was measured with the photon-counting pixel detector.

We extracted the profiles of the integrated *I*_XBIC_ in the axial (*z*-axis) and radial (*y*-axis) directions of the nanowire, shown in Figs. 4 ▸(*c*) and 4(*e*), respectively. The axial *I*_XBIC_ profile in Fig. 4 ▸(*c*) is similar to our previous report (Chayanun *et al.*, 2019*b* ▸). The characteristic decay length of the slopes on both sides (left ∼500 nm, and right ∼120 nm) of these profiles corresponds to the charge transport within the nanowires (Mohite *et al.*, 2012 ▸; Gutsche *et al.*, 2012 ▸). The longer decay on the left slope is the result of the gradient p–i junction caused by the memory effect during the nanowire growth (Chayanun *et al.*, 2019*b* ▸). Our previous investigations analyzed these axial profiles in detail, while the spatial resolution was insufficient for the radial direction. Here, we can reveal *I*_XBIC_ profiles in the radial direction of the nanowire with several data points in Fig. 4 ▸(*c*). These radial profiles at different Φ were fitted with the Gaussian distribution function in which their full width at half-maximum (FWHM_XBIC_) almost linearly increases with *I*_XBIC_ (see supporting information). The measured FWHM_XBIC_ are lower than the nanowire diameter, which may seem unreasonable. However, the XBIC signal is reduced by secondary photons and electrons that escape the sample (Chayanun *et al.*, 2019*b* ▸; Stuckelberger *et al.*, 2015 ▸), and this effect is stronger near the surface of the nanowire.

The XBIC signal depends on the local electric field, which can be systematically varied by applying an external bias (Chayanun *et al.*, 2019*b* ▸). Therefore, we performed bias-dependent XBIC measurements using Φ = 4.14 × 10^6^ photons s^−1^ (*T* = 17.2%). Figure 4 ▸(*d*) shows the XBIC maps of the nanowire, at applied biases ranging from −0.5 V to 0.4 V, while Fig. 4 ▸(*e*) shows the axial and radial profiles. Generally, *I*_XBIC_ increased from forward bias to reverse bias, similar to our previous investigations (Chayanun *et al.*, 2019*b* ▸). The maximum *I*_XBIC_ saturates at negative bias, as is most clearly observed in the axial and radial *I*_XBIC_ profiles in Fig. 4 ▸(*e*). The reverse bias enhances the built-in electric field in the diode, and it becomes so strong that all generated carriers are collected. The opposite effect is observed with forward bias, where the reduced electric field in the depletion region leads to a lower charge collection efficiency (CCE). Moreover, the XBIC area is reduced along the axial direction and shifted toward the n-segment [see Fig. 4 ▸(*d*)]. Measurements using XRF (Troian *et al.*, 2018 ▸) as well as the computer simulations (Chayanun *et al.*, 2019*b* ▸) show that an unintentional p-doping in the middle segment causes this asymmetric change of the XBIC area. Here, we can observe a complete two-dimensional charge collection map of a single nanowire using the high-resolution MZP optics.

As a comparison, XBIC scans using a fully illuminated MZP without a central stop and an OSA can be found in the supporting information. The measurements using the off-axis illuminated MZP benefit from a constant background independent of the applied voltage, absent of background photons, which allows the use of a uniform color scaling. With the fully illuminated MZP without a central stop and an OSA, the background signal is changing between the scans. Further, despite the nominally higher resolution of a fully illuminated MZP, the corresponding XBIC maps of the nanowire have a lower resolution due to photons coming from different diffraction orders (see supporting information).

### 3.4. Mapping the MZP focus with the nanowire device   

**Figure 5:**
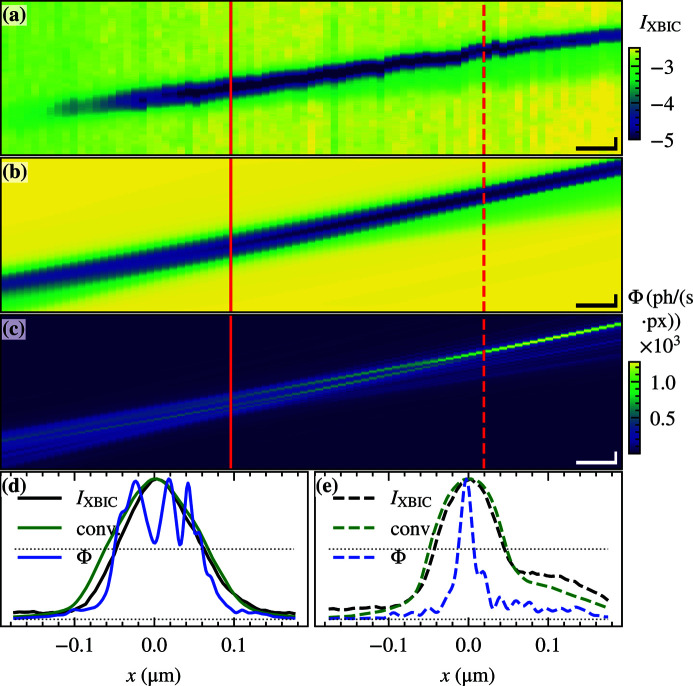
Comparison of the X-ray beam path using XBIC and ptychography. (*a*) XBIC measurement of an off-axis illuminated MZP with apertures. (*b*) Convolution of the ptychographically reconstructed probe with a nanowire profile. (*c*) The corresponding ptychographic reconstruction. The dashed line is the plane of focus; the solid line is the plane of measurement. (*d*) Line profile in the focus plane and (*e*) the measurement plane. Scalebars (*a*–*c*) vertical 100 nm, horizontal 10 µm, (*d*,*e*) 100 nm

Nanowire diodes are sufficiently small to be used as single-pixel detectors at a far better resolution than conventional detectors. We have previously used nanowire devices to map the KB mirror focus at the P10-GINIX (Wallentin *et al.*, 2014 ▸) and the NanoMax (Chayanun *et al.*, 2020 ▸) beamlines. Here, the nanowire device was used to map the MZP beam path by using it as a 1D detector, in the direction across the nanowire. Figure 5 ▸(*a*) shows the beam path of the focused X-rays along the *z*-axis (propagation direction) and *x*-axis (horizontal plane). The map was recorded by scanning a vertical nanowire along both axes and performed using the off-axis illuminated MZP. The illumination time was 0.1 s, the step size in the *z*-direction was 2 µm and the step size in the *x*-direction was 10 nm again in fly-scan operation. An equivalent measurement using a fully illuminated MZP without a central stop and an OSA can be found in the supporting information. The XBIC measurements of the beam path focused by the off-axis illuminated MZP resolves the divergent angle of the beam relative to the beamline orientation, the rising intensity in the vicinity of the focus and a small side maximum in the focal plane. Also, it shows a decrease in the XBIC signal on the left side where the nanowire is no longer in the focal plane. Further, along the beam path, small movements of approximately 20 nm of the beam relative to the nanowire can be observed.

The measured XBIC line profile can be calculated as the convolution of the nanowire profile with the X-ray beam. Figure 5 ▸(*b*) shows the calculated convolution for a 180 nm nanowire profile with the ptychographically reconstructed X-ray beam shown in Fig. 3 ▸(*c*). The XBIC measurement and the convolution are in good agreement. The convolution shows a divergence and a rising side maximum which is also apparent in the XBIC measurement. A 2 mrad difference in the beam path angle can be related to a misalignment of the motor *x*-axis relative to the beam. The corresponding beam profile in the ptychographic focal plane is shown in Fig. 5 ▸(*e*). Nevertheless, the limit in resolution of the nanowire detector with respect to the very small focus from the MZP becomes apparent, which makes it challenging to determine the focal plane using only the XBIC measurements. The comparison of the XBIC measurement and the subsequently performed ptychographic measurement shows that the nanowires were not measured in the optimal focal plane, but at a distance of 64 µm from it. In Fig. 5 ▸(*d*) the beam profile in this plane is shown with a width of 192 nm based on the ptychographic reconstruction. This corresponds to the width of the nanowire (180 nm) and its resolution threshold, being the reason for the incorrectly chosen measurement plane.

Although ptychography provides higher resolution in determining the probe, several constraints must be met for a successful reconstruction, such as coherence, compactness, sampling and high stability. As a result, ptychography is limited to dedicated setups and beamlines. In contrast, the XBIC method measures photons directly in the object plane. In the present case, the orientation of the nanowire led to a limited spatial resolution and a weak signal. We have recently demonstrated that much better spatial resolution can be achieved by orienting the nanowire parallel to the beam (Chayanun *et al.*, 2020 ▸). This increases the resolution to 60 nm together with the photon sensitivity, since the absorption length is now given by the nanowire length rather than the diameter.

## 4. Conclusion and outlook   

We have presented a new approach to implement a 2D focusing optics in an off-axis geometry, with the main advantage of more than doubling the useful working distance to the sample. The focus size of the off-axis illuminated MZP was determined with a FWHM of 16.2 nm × 27.9 nm. Even though the focus is larger than that of a fully illuminated MZP (8.4 nm × 9.6 nm), the increased distance to the nearest OSA is what makes it possible to perform experiments with complex samples in the focal plane in the first place. Thereby experiments to measure the XRF, STXM or XBIC signal with a small focus size and with a low background signal become possible. MZPs (and MLLs) have a very short focal depth, whether classic or off-axis, which makes optimal sample alignment challenging. Our results show that ‘real-time’ ptychographic measurements can be very helpful in sample alignment.

In addition, the focus of an off-axis illuminated MZP can be further reduced if the area of illumination and thereby the NA is increased. In the case of the experiment performed, a large part of the MZP was not illuminated and therefore a large part of the possible NA was not used. An illumination of ellipsoidal shape using a larger area could increase the NA and would still result in a separation of the focused beam and the initial beam axis. However, to gain the full benefits of an off-axis illuminated MZP, specially designed off-axis MZPs should be fabricated. A specially designed off-axis MZP would have the advantage that the area which needs to be fabricated is reduced to only one side of the MZP. This enables the fabrication of larger optics with a smaller focal spot size, or alternatively a larger focal length. An example simulation of a dedicated off-axis MZP with a focal length of 1.84 mm is provided in the supporting information. The size of the resulting focal spot is 9.0 nm × 10.5 nm, whereby fabrication details are similar to current specifications, especially with regard to total size and outermost zone width.

The focused off-axis beam was used to perform a mapping of the charge carrier distribution using XBIC. The measurements benefited from a low background signal. We were able to perform for the first time a 2D mapping of the area of InP nanowires charge carrier collection under various flux and applied bias settings (see Fig. 4 ▸). By optimizing the MZP optics and sample alignment, it should become feasible to acquire XBIC maps at a spatial resolution of less than 10 nm. This is substantially better than electron-beam methods, which suffer from an inherent broadening due to electron–electron scattering (Stuckelberger *et al.*, 2015 ▸). The low background of the off-axis MZP approach also makes it highly suitable for X-ray fluorescence, X-ray diffraction and STXM, signals that in principle can be acquired simultaneously (Chayanun *et al.*, 2019*a* ▸). The longer working distance could also allow more complex *in situ* and *operando* studies. The off-axis MZP is well suited for the current upgrades of synchrotron radiation sources to diffraction-limited storage rings. Altogether, this opens a brilliant perspective for ultrahigh-resolution multimode imaging of nanostructures.

## 5. Supporting information   

Supporting information including ptychographic reconstructions, finite difference simulations, analysis of the flux dependence of the XBIC signal, and an XBIC mapping using a fully illuminated MZP can be found in the online supporting information.

## 6. Related literature   

The following references, not cited in the main body of the paper, have been cited in the supporting information: Alig & Bloom (1978 ▸); Miao *et al.* (1998 ▸); Salditt *et al.* (2015 ▸); Yan *et al.* (2014 ▸); Zozulya *et al.* (2012 ▸).

## Supplementary Material

Sections S1 to S9; Figures S1 to S10; Table S1. DOI: 10.1107/S1600577521006159/gb5120sup1.pdf

## Figures and Tables

**Table 1:** Basic parameters of the two beamline endstations where the experiments were performed The distance of the detector to the MZP is defined as Δ_Detector_. The focal length of the MZP is defined as *f*_MZP_ and the maximum distance between the OSA and the focus of the MZP is defined as Δ_OSA–focus_. The size of the OSA is given by *D*_OSA_.

Beamline	*E*	Monochromator	Pre-focus	Δ_Detector_	*f*_MZP_	Δ_OSA–focus_	*D*_OSA_
P10-GINIX	13.8 keV	Si(111) pair channel-cut	CRL	5.10 m	0.92 mm	0.44 mm	3.5 µm
P06-Nanoprobe	15.0 keV	Si(111) pair channel-cut	CRL	3.43 m	1.00 mm	0.18 mm	3 µm
