# Supplementary material for: Off-axis multilayer zone plate with 16 nm × 28 nm focus for high-resolution X-ray beam induced current imaging
Source: J Synchrotron Radiat. 2021 Jul 22;28(Pt 5):1573–82. doi: 10.1107/S1600577521006159 (PMC8415331; doi:10.1107/S1600577521006159)
Supplement: Supplementary file 1 [file s-28-01573-sup1.pdf]

Supporting Information: Off-axis Multilayer Zone Plate with $16 \times 28\,\text{nm}^2$ focus for high resolution X-ray Beam Induced Current imaging

Jakob Soltau,[a] Lert Chayanun,[b] Mikhail Lyubomirskiy,[c]
Jesper Wallentin[b] and Markus Osterhoff[a]

[a]*Institute for X-ray physics, University of Göttingen, Friedrich-Hund-Platz 1, Göttingen, 37077, Germany,* [b]*Synchrotron Radiation Research and NanoLund, Lund University, Box 118, Lund 22100, Sweden, and* [c]*Deutsches Elektronen-Synchrotron DESY, Notkestrasse 85, 22607 Hamburg, Germany*

1. P10-GINIX setup

The experiment using the off-axis illuminated MZP was performed at the Göttingen instrument of nano-imaging with X-rays (GINIX) at the coherence beamline P10 of the PETRA III storage ring (Hamburg, Germany) (Salditt *et al.*, 2015). The beamline uses a 5 m long undulator positioned at a distance of 88.5 m from the experimental hutch. In the described experiment the undulator beam was monochromatized to 13.8 keV using a Si(111) channel-cut monochromator. The monochromator is positioned at a distance of 50 m from the experimental hutch. Using compound refractive lenses (CRL) installed in an ultra-high vacuum chamber in the experimental hutch the beam is pre-focused to about 16 µm in horizontal and vertical direction. The focal length of the used CRLs is about 1.9 m. A detailed analysis of the CRL efficiencies can be found in (Zozulya *et al.*, 2012). The MZP and the nanowires are mounted on

the high-resolution stage of the GINIX instrument. The sample stage is motorized using piezo scanner by Physik Instrumente Karlsruhe (PI), the MZP stage by stick-slip positioners by SmarAct (Oldenburg) for translations, and a piezo-driven Gimbal mount for rotations. For detailed information on the stability and scanning precision see (Osterhoff *et al.*, 2017). The pinhole and the OSA were mounted on piezo scanners by SmarAct (Oldenburg). The diameter of the pinhole was 5.6 µm, the diameter of the OSA was 3.5 µm. The distance of the OSA relative to the focus was 0.44 mm.

At a distance of 5.1 m behind the sample the detector was positioned. A single photon counting Eiger 4m (Dectris Ltd., Switzerland) detector was used with 2068 × 2162 pixels with a pixel size of 75 µm.

2. P06-Nanoprobe setup

The experiment using the fully illuminated MZP was performed at the PtyNAMI instrument (Schropp *et al.*, 2020) of the Hard X-ray Micro/Nano-Probe at the beamline P06, which is positioned at the PETRA III storage ring. The beamline uses a 2 m long undulator positioned at a distance of 97.5 m from the nanohutch where the experiment was preformed. The undulator beam was monochromatized using a Si(111) channel-cut monochromator to 15 keV. The monochromator is positioned at a distance of 59.1 m from the nanohutch. The distance of the CRLs to the nanohutch is 54 m. Using the PtyNAMI instrument the pinhole, OSA, MZP and sample were mounted on piezo stages by SmarAct (Oldenburg). The diameter of the central stop was 6 µm, the diameter of the OSA was 3 µm. The distance of the OSA relative to the focus was 0.25 mm.

The diffraction patterns were recorded at a distance of 3.43 m relative to the sample using a single photon counting pixel detector (Pilatus 300k, Dectris Ltd. Switzerland). The detector has 619 × 487 pixels and a pixel size of $\Delta_{\text{px}} = 172$ µm.

3. Ptychographic reconstructions

3.1. Full Illuminated MZP

The probe of the full illuminated MZP was characterized using ptychography. The measurement was performed at the P06-Nanoprobe beamline (Schroer *et al.*, 2016), as described in the manuscript. A scan was performed with 25×51 scan points and an illumination time per frame of 0.2 s. As a sample a Siemens star was used with a smallest feature size of 50 nm. The Siemens star was positioned at a distance of 70 µm relative to the focus. For the reconstruction the ptychography script of the beamline was used. The script is based on the ePIE algorithm (Maiden & Rodenburg, 2009). In Fig. 1 the results of the ptychographic reconstruction are depicted. In (a) the absorption of the object is shown, in (b) the phase of the object and in (c) the probe in the object plane. The smallest features of the object are well resolved. Further sub-structures are visible in the center of the Siemens star. These sub-structures are not an artifact from the reconstruction process, but real and due to beam damage resulting from a previous experiment.

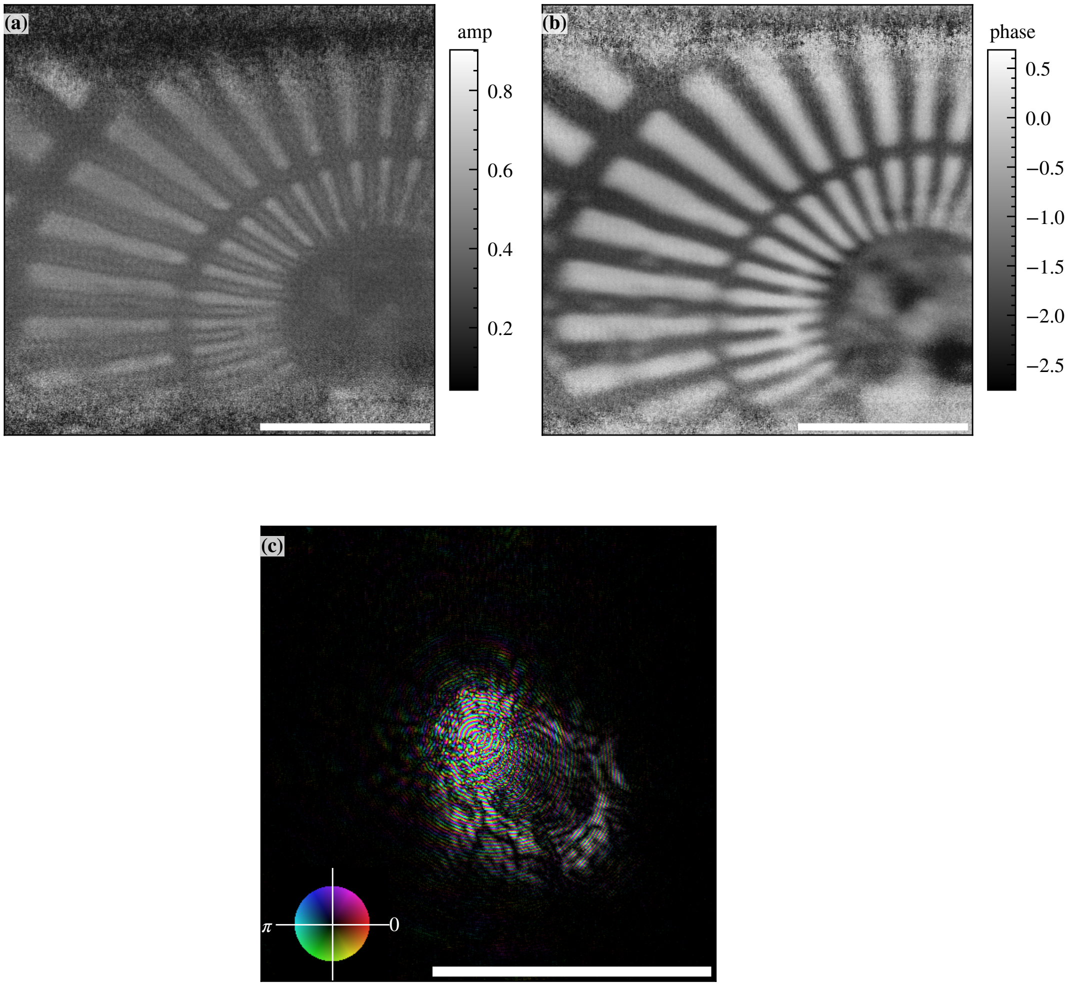

Fig. 1. Ptychography using a Probe focused by a full illuminated MZP. (a) Object amplitude, (b) object phase, (c) probe. Scalebars: 1 μm

3.2. Off-Axis Illuminated MZP

The probe of the off-axis illuminated MZP was characterized using ptychography. The measurement was performed at the P10-GINIX beamline (Kalbfleisch *et al.*, 2011), as described in the manuscript. A scan was performed with 41×41 scan points and an illumination time per frame of 1.0 s. A lithographic test pattern with smallest structure size of 100 nm was used as a sample. The sample was positioned at a

distance of 350 µm relative to the focus. The diameter of the beam in this plane was about 1.75 µm^2. For reconstruction our own ptychographic script was used. The script is based on the ePIE algorithm (Maiden & Rodenburg, 2009). In Fig. 2 the results of the ptychographic reconstruction are depicted. In (a) the absorption of the object is shown, in (b) the phase of the object and in (c) the probe in the object plane. The smallest features of the object are well resolved. The shape of the probe is quiet similar to the shape measured at the detector and is due to the relative large distance between focus and object.

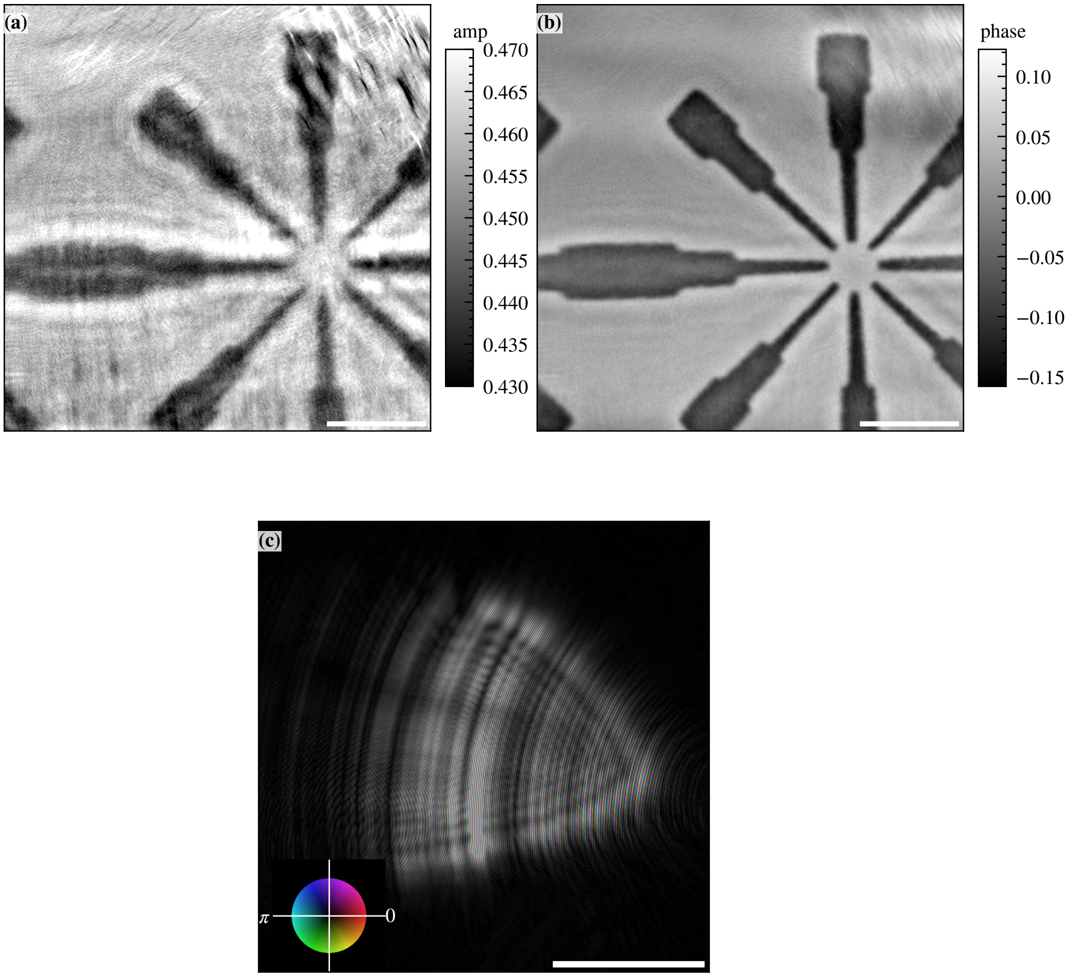

Fig. 2. Ptychography with probe focused by an off-axis illuminated MZP. (a) Object amplitude, (b) object phase, (c) probe. Scalebars: 1 µm

4. Finite Differences Simulations

Simulations were performed to estimate the focusing properties of an MZP with the exact specifications as the one used in the experiments at the P10-GINIX instrument. The parameters of the simulated MZP were: 0.92 mm focal length, 784 zones, first inner zone number 14 (equivalent to a diameter of 2.1 µm) 5 nm outermost zone width, 15.6 µm radial size, 2.4 µm optical thickness and a tilting angle of 2.5 mrad. The energy

of the illumination was 13.8 keV. The simulations were based on a finite differences (FD) solver (Melchior & Salditt, 2017). To account for the circular shape of the MZP the simulations were performed in three dimensions. The numerical grid parameters were $\Delta_{x,y} = 1\,\mathrm{nm}$ and $\Delta_z = 10\,\mathrm{nm}$ for the lateral and the propagation directions, respectively. In Fig. 3(a) the beam profile of an MZP fully illuminated by a plane wave is shown. No apertures (central stop and OSA) was simulated. This results in a background signal by the non-diffracted photons. In the inset the lateral extension of the focus is shown. The FWHM of the focus is $5.9 \times 5.9\,\mathrm{nm}^2$. This is inline with the smallest outermost zone size of 5 nm. In Fig. 3(b) the far-field diffraction pattern can be seen. In the far-field pattern the non-diffracted photons are in the center pixels. The photons diffracted in the first positive and negative orders are distributed over the entire far-field. The diffraction patterns of both orders overlay each other and are therefore indistinguishable.

Next, the illumination of the MZP by an off-axis beam was simulated in the same configuration. The initial beam has a Gaussian shape with an FWHM of 4 µm and a lateral off-axis position of 6 µm. The propagation of the beam behind the MZP is shown in Fig. 3(c). Again no apertures (pinhole and OSA) were simulated. In the inset the focus extension in lateral dimension is shown. The FWHM of the focus is $12.1 \times 15.4\,\mathrm{nm}^2$. The separation of the focus and the non-diffracted beam can be seen. A sample which would be positioned in the focus would not be penetrated by background photons. In Fig. 3(d) the far-field diffraction pattern of the simulated off-axis beam is shown. The photons diffracted in the plus first order are in the lower part, the photons of the negative first diffraction order are in the upper part and in the center the non-diffracted photons. In an actual experiment the photons of the non-diffracted beam and the first negative order would not be visible due to the positioning of the OSA. The difference in the size of the area of the photons diffracted in the negative

and positive order, is due to the tilting of the individual zones and therefore different diffraction efficiencies.

Whereas the simulations in Fig. 3 are based on the specifications of an MZP which was already fabricated, is a potential dedicated off-axis MZP shown in Fig. 4. Since the total deposition process stability is the bottle neck in the fabrication of larger MZPs, the fabrication of an MZP with larger focal length is challenging. But by fabricating an MZP with zones on only one side, the deposition time per zone could be decreased and thus the total number of zones increased. The dedicated off-axis MZP simulated here has the following parameters: 1568 zones (twice the number of zones as produced so far), 1.84 mm focal length, 5 nm outermost zone width. The size of the Gaussian shaped illumination was 15 µm with an off-axis position of 12 µm. The simulated beam path is shown in Fig. 4 with a focus FWHM of $9.0 \times 10.5\,\mathrm{nm}^2$ (see inset) positioned 1.84 mm behind the MZP, which is twice the length as the MZPs used in the described XBIC experiments. Conversely, it would also be possible to maintain the focal length but reduce the focus size.

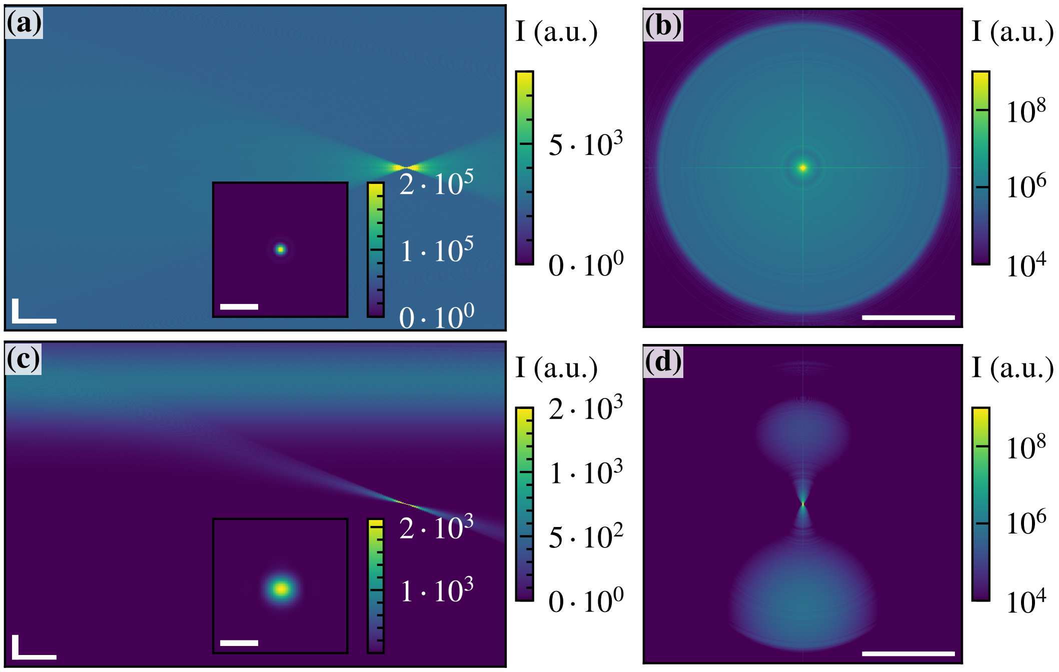

Fig. 3. Results of FD-simulations in three dimensions. (a) Shows the propagating wave field behind a full illuminated MZP. At a distance of 1 mm the focus can be seen. The non-diffracted photons are generating a background signal. The inset shows the focal spot in its lateral extension. The focus has a FWHM of $5.9 \times 5.9\,\text{nm}^2$. The corresponding far-field is shown in (b). (c) shows the propagating wave field behind an MZP illuminated by an off-axis Gaussian shaped beam. The focus at a distance of also 1 mm is clearly separated from the non-diffracted photons. The focus has a FWHM of $12.1 \times 15.4\,\text{nm}^2$ and is shown in its lateral extension in the inset of the figure. The corresponding far-field is shown in (d). The positive and negative orders are separated and not overlapping. Scalebars: (a,c) vertical 100 µm and horizontal 1 µm, the insets 25 nm (b,d) $q = 0.26\,\text{nm}^{-1}$

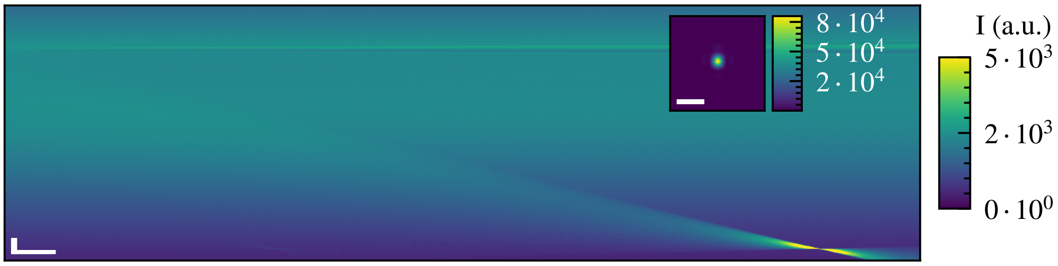

Fig. 4. Beam path simulation of a dedicated off-axis MZP with 1.84 mm focal length. This off-axis MZP has twice the number of zones compared to the MZP used in the experiment but is only one sided. The inset shows the lateral extension of the focus with $9 \times 10.5\,\mathrm{nm}^2$. Scalebars: vertical 100 µm and horizontal 1 µm, the inset 25 nm

5. Side maxima when center part is blocked

Figure 5 compares the height of the side maxima of a focus from a full illuminated MZP with central stop and an off-axis illuminated MZP which has the same NA. As described by (Simpson & Michette, 1984) for the case of round diffractive optics, focus side maxima increase the more zones in the center are not illuminated. Fig. 5 (a) shows the simulated focus of a MZP with the following parameters: photon energy 13.8 keV, focal length 0.92 mm, optical depth 2.5 µm, MZP diameter 16.5 µm, outer most layer width 5 nm, center stop diameter 8.24 µm. All layers are tilted according to the wedge geometry (Yan *et al.*, 2014). Fig. 5(b) shows the focus of an off-axis MZP with the same off-axis gap and the same diameter as the simulated MZP shown in (a). The only difference between the simulated MZP in (a) and (b) is the outer most layer width which is in case of (b) only 2 nm. This is due to the requirement for the simulation that both optics should have the same NA. The simulation were performed using the FD algorithm. In case of (a) the propagation of the field was simulated in a radial coordinate system (Melchior & Salditt, 2017), propagation step size was 10 nm and the lateral grid size was 0.1 nm. In case of (b) an 2D Cartesian coordinate system

was used to simulate the propagation of the off-axis geometry. The simulation grid sizes were the same as in (a). The normalized focus profiles from (a) and (b) are shown in (c) for comparison. In case of the full illuminated MZP with a central stop the side maxima have a height of 10.6 % of the maximum intensity of the focus, in contrast the side maxima peak height for the off-axis illuminated MZP is only 4.5 % of the maximum intensity. This result in a relative difference of the normalized side maxima by a factor of 2.31 between the off-axis illuminated MZP and the full illuminated MZP. The large side maxima result in a reduced effective resolution of the focusing optic. Additionally, also the FWHM of the main focus is slightly smaller for the case of the off-axis illuminated MZP.

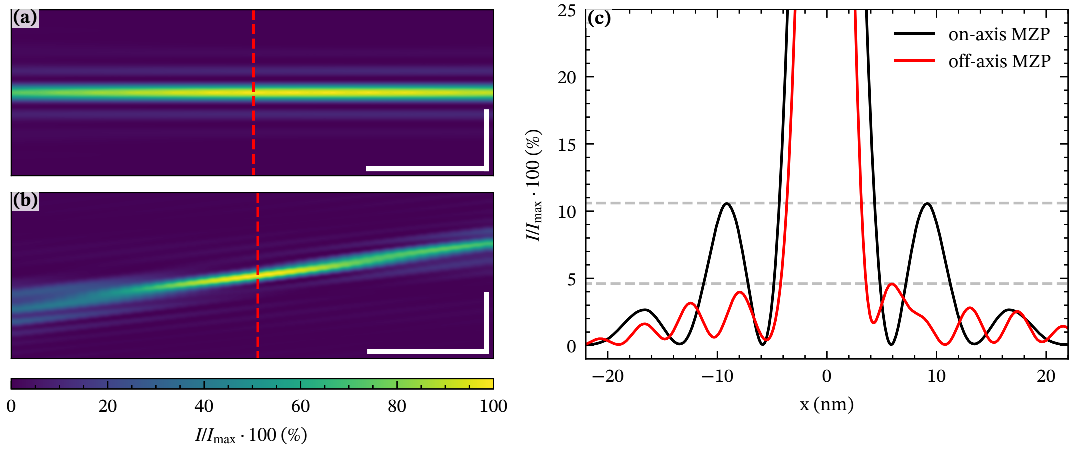

Fig. 5. Comparison of the focus side maxima for the case of (a) a full on-axis illuminated MZP with central stop and (b) an off-axis illuminated MZP. The NA of both configurations is the same with an illuminated area of 16.5 µm and a focal length of 0.92 mm at a photon energy of 13.8 keV. (c) the normalized intensity in the focal plane is plotted. In (a) and (b) the focal plane is indicated by the red dotted line. In (c) the height of the side maxima is indicated by the gray dotted line. The difference in the height is a factor of 2.3. Scalebars: (a,b) vertical 25 nm and horizontal 500 nm

6. Simulations of flat and tilted MZPs

Figure 6 shows the focal points of a flat MZP and the focal point of a tilted MZP. The intensity is normalized to the maximum intensity of the titled MZP. The focus was simulated using FD in a circular coordinate system (Melchior & Salditt, 2017). The parameters of the simulated MZPs were: 0.92 mm focal length, 784 zones, 5 nm outermost zone width, 15.6 µm radial size, 2.5 µm optical thickness. The energy of the illumination was 13.8 keV. For the MZP in tilted geometry a tilting angle of 2.5 mrad was assumed. In Fig. 6(a,b) the intensity distributions of both focal points is shown with the same color scaling. The difference of the intensity of the focal spots is a factor of 2.9 as can be seen in the plotted focus profiles shown in Fig. 6(c). This demonstrates the improved focusing efficiency of a MZP based on the tilted geometry compared to a MZP in a flat geometry.

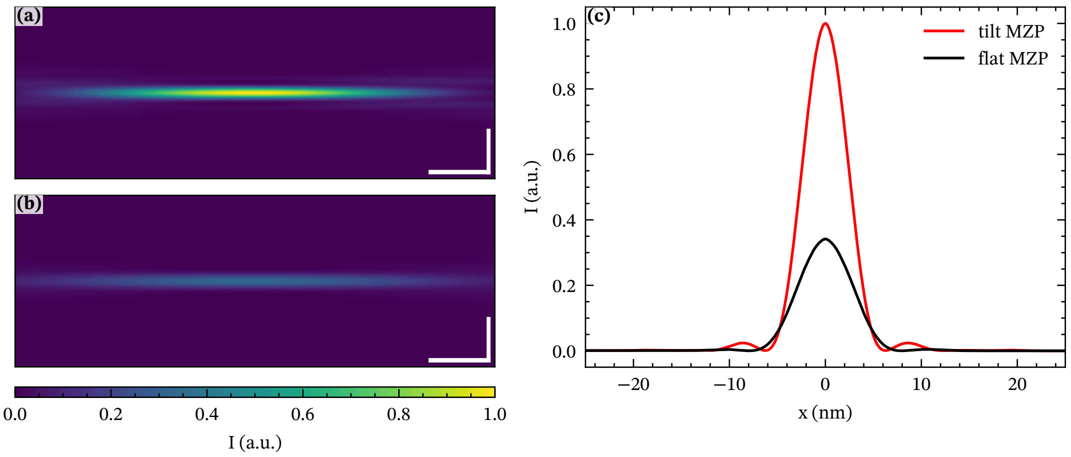

Fig. 6. Comparison of the focusing efficiency of a MZP in tilted and a MZP in flat geometry using FD-simulations. (a) shows the intensity distribution of the focus of a tilted MZP. (b) shows the equivalent intensity distribution of a flat MZP. In (a) and (b) the intensity is normalized by the peak intensity of the tilted MZP. (c) shows a the focus profile of the flat and the tilted MZP. The maximum intensity of the flat and tilted MZP differ by a factor of 2.9. Scalebars: (a,b) vertical 25 nm and horizontal 0.5 µm

7. Comparison of on- and off-axis illuminated MZPs

Table 1. *Comparison of different central stop configurations of fully illuminated MZPs and configurations of off-axis illuminated MZPs. The NA of* $8.7 \cdot 10^{-3}$ *is equivalent to the fully illuminated MZP used for the experiments at* 13.8 keV *photon energy at the GINIX-P10 setup.* D_{CS} *is the size of the center stop,* R_{off} *is the radial off-axis position of the off-axis MZP.* z_{WD} *is the working distance between the focus and the OSA.* A_{MZP}/A_{CS} *is the area of the fully illuminated MZP which is covered by the central stop. In case of the off-axis illuminated MZP no central stop is required and therefore no area is covered. The focus FWHM* d_{FWHM} *was simulated using finite differences. It should be noted that additional parameters such as the intensity of the side maxima or in case of the on-axis illuminated MZPs with a central stop the flux density distribution, which is in most case of Gaussian shape, have an additional effect in selecting the best possible configuration for an experiment.*

	MZP		off-axis MZP	
NA	$8.7 \cdot 10^{-3}$	$8.7 \cdot 10^{-3}$	$4.4 \cdot 10^{-3}$	$8.7 \cdot 10^{-3}$
D_{CS}	6 µm	12 µm	-	-
A_{MZP}/A_{CS}	86 %	44 %	-	-
R_{off}	-	-	6 µm	6 µm
z_{WD}	197 µm	276 µm	394 µm	251 µm
d_{FWHM}	4.8 nm	4.1 nm	8.0 nm	4.3 nm

8. Flux dependence of the XBIC signal

In Fig.7 the maximum I_{XBIC} is plotted against the photon flux Φ, where the expected linear relation can be observed. Theoretically, I_{XBIC} can be estimated using the equation, $I_{\mathrm{XBIC}} = q\eta p_{abs}\Phi S\left(x,y,z\right)$, where q is the charge constant, η is the charge generation yield, $p_{|\Phi S(x,y,z)|}$ is the x-ray absorption probability, and $S\left(x,y,z\right)$ is the relative spatially dependent charge collection efficiency (Chayanun *et al.*, 2019). The charge generation yield is the ratio between the energy of the x-rays, E, and the ionization energy of the semiconductor ϵ, so that $\eta = E/\epsilon$ (Alig & Bloom, 1978). In the case of a very thin sample, p_{abs} can be approximated from $p_{abs} = \mu d$, where μ is the absorption coefficient, and d is the thickness of the sample. At the maximum I_{XBIC} from the map, we can assume the maximum charge collection efficiency, and therefore $S\left(x,y,z\right) = 1$ (Chayanun *et al.*, 2019). Consequently, I_{XBIC} as a function

of Φ can be written as $I_{\mathrm{XBIC}} = 1.7062 \times 10^{-18} \cdot \Phi$ for the x-ray energy of 13.8 keV. This function is plotted in Fig.7 as a red line. We can observe the difference between the maximum measured and the theoretically calculated I_{XBIC} from the plot in Fig. 7. This low measured XBIC comparing to the calculation was evidenced before in the previous publication. In this experiment, the measured I_{XBIC} is about 20 % of the theoretical calculation, which could attribute to the escaping of those secondary electrons from this nanostructure sample (Chayanun *et al.*, 2019; Stuckelberger *et al.*, 2017). Hence, we introduced the term for the new charge generation yield of the nanowire compensating the actual yield with the escaping secondary charges.

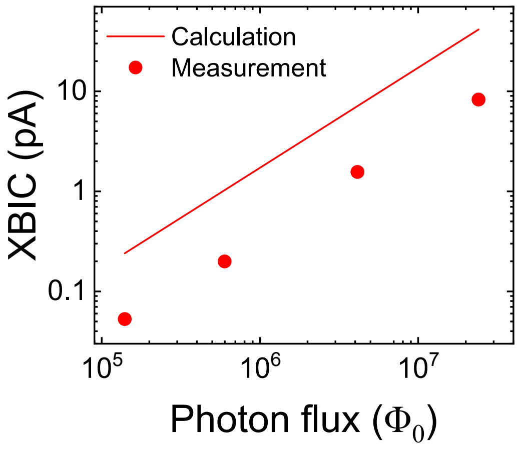

Fig. 7. The plot of the maximum I_{XBIC} (red circle) and the calculated I_{XBIC} (red line) against the x-ray photon flux.

In Fig.8 the radially profiles at different X-ray photon fluxes and bias-dependent XBIC measurements are compared. The profiles are fitted using the Gaussian distribution function and the full-width-half-maximum (FWHM) is defined. The FWHM increases almost linearly with the X-ray photon flux, respectively the XBIC signal. The same applies to the bias-dependent measurements.

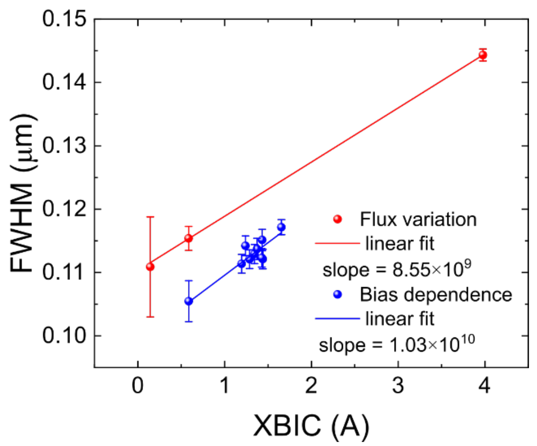

Fig. 8. Comparisons of the different radial XBIC profiles. The FWHM of the radial XBIC profiles is plotted against the maximum XBIC signal from the X-ray photon flux variation and the bias-dependent XBIC measurements. The difference between the two measurements can be explained by variations in the intensity of the X-ray beam.

9. XBIC by a Full Illuminated MZP

9.1. Bias dependent measurements

Beside the XBIC measurements of the off-axis illuminated MZP described in the manuscript, measurements using a full-illuminated MZP at the P10 beamline were performed as well. Therefore the OSA (and the pinhole) were moved out of the beam, since the nanowire device needs more free space in the vicinity of the focus. For a fully illuminated MZP the maximum free space between the focus/sample and the OSA would be below 180 µm, which is not enough for the positioning of the nanowire device.

Equivalent to the bias dependent measurements presented in the manuscript using an off-axis illuminated MZP, bias dependent measurements were performed using the full illuminated MZP. The XBIC maps at different applied biases ranging from -0.5 V to 0.4 V with the increment of 0.1 V and are shown in Fig. 9. The scan was done with 20 nm step size and 0.1 s acquisition time. The I_{XBIC} presents the charge collection

of the device. Just like in the figure in the main manuscript the range of the color bar is the same for all maps. For the full illuminated MZP, this results in a bias dependent background signal. This is different for the measurements using the off-axis illuminated MZP presented in the main manuscript, where the color bar range is equal for all maps, and no bias-dependent background signal was observed.

Furthermore, in the case of the fully illuminated MZP, the shape of the nanowire XBIC signal, although similar, is much broader. This is contradictory at first, since the fully illuminated MZP produces a smaller focus. However this is due to the photons from different diffraction orders which also produce an XBIC signal, which then leads to the broadening of the nanowire signal.

This demonstrates for the case of the XBIC measurement in the described experiment, that the slightly larger focus of the off-axis MZP with OSAs and therefore negligible background photons is resulting in a better measurement as the smaller focus of the full illuminated MZP without OSAs and therefore with a background signal.

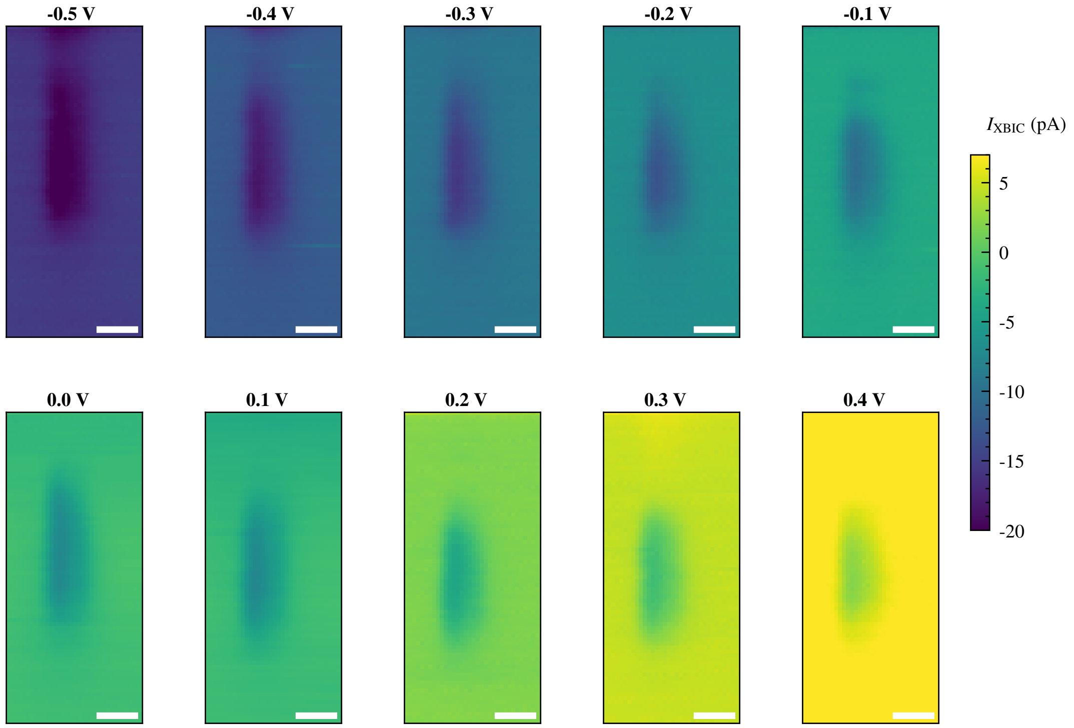

Fig. 9. Voltage variation from −0.5 V to 0.4 V with the increment of 0.1 V using the full illuminated MZP with no apertures (central stop and OSA). Scalebars: 100 nm

9.2. Measurement of the Beam path

Equivalent to the beam path characterization by using the nanowire device as an x-ray detector in the manuscript a second scan was performed using the full illuminated MZP again with no apertures (central stop and OSA). The measured XBIC signal of the focused beam is shown in Fig. 10. Only few photons are detected in the region of the center, where the first zones are missing due to the glass wire. Similar to the measurement of the off-axis MZP a small miss alignment (≈ 0.6 mrad) of the motor-axis relative to the propagation direction of the beam can be seen. Most photons are detected along the diffraction angles of 4.8 mrad. According to literature the best diffraction efficiency in the direction of the focus is achieved when the zones are tilted

by half the diffraction angle (Yan *et al.*, 2010). This is consistent with the results measured with the nanowire instrument, as the zones of the MZP are tilted by $\approx$ 2.5 mrad.

From the XBIC measurement it is challenging to determine the size of the focus because first the background photons are generating an XBIC signal as well and second as mentioned in the manuscript the resolution of the nanowire device as a detector is limited by the width of the nanowire (180 nm). It should be noted that the measurement already gives a good impression of the beam path and reveals the position of the focal plane. In contrast, in this setup configuration a comparative determination of the beam path by using a ptychography is not possible due to the non-existence of a central stop and an OSA and therefore an unfulfilled sampling constraint (Miao *et al.*, 1998).

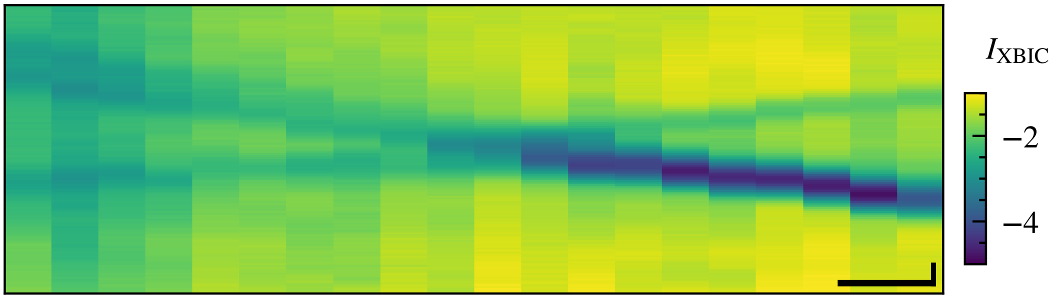

Fig. 10. XBIC measurements of the beam path of a full illuminated MZP with no apertures (central stop and OSA). Scalebar: vertical 100 nm, horizontal 10 µm

References

Alig, R. & Bloom, S. (1978). *Journal of Applied Physics*, **49**(6), 3476–3480.

Chayanun, L., Otnes, G., Troian, A., Hammarberg, S., Salomon, D., Borgström, M. T. & Wallentin, J. (2019). *Journal of synchrotron radiation*, **26**(1), 102–108.

Kalbfleisch, S., Neubauer, H., Krüger, S. P., Bartels, M., Osterhoff, M., Mai, D. D., Giewekemeyer, K., Hartmann, B., Sprung, M. & Salditt, T. (2011). *AIP Conference Proceedings*, **1365**(1), 96–99.
URL: *https://aip.scitation.org/doi/abs/10.1063/1.3625313*

Maiden, A. M. & Rodenburg, J. M. (2009). *Ultramicroscopy*, **109**(10), 1256–1262.

Melchior, L. & Salditt, T. (2017). *Optics Express*, **25**(25), 32090–32109.

Miao, J., Sayre, D. & Chapman, H. (1998). *JOSA A*, **15**(6), 1662–1669.

Osterhoff, M., Eberl, C., Soltau, J. & Krebs, H.-U. (2017). In *Journal of Physics: Conference Series*, vol. 849, p. 012049. IOP Publishing.

Salditt, T., Osterhoff, M., Krenkel, M., Wilke, R. N., Priebe, M., Bartels, M., Kalbfleisch, S. & Sprung, M. (2015). *Journal of synchrotron radiation*, **22**(4), 867–878.

Schroer, C. G., Baumbach, C., Döhrmann, R., Klare, S., Hoppe, R., Kahnt, M., Patommel, J., Reinhardt, J., Ritter, S., Samberg, D., Scholz, M., Schropp, A., Seiboth, F., Seyrich, M., Wittwer, F. & Falkenberg, G. (2016). *AIP Conference Proceedings*, **1741**(1), 030007. **URL:** *https://aip.scitation.org/doi/abs/10.1063/1.4952830*

Schropp, A., Döhrmann, R., Botta, S., Brückner, D., Kahnt, M., Lyubomirskiy, M., Ossig, C., Scholz, M., Seyrich, M., Stuckelberger, M. E. *et al.* (2020). *Journal of applied crystallography*, **53**(4), 957–971.

Simpson, M. & Michette, A. (1984). *Optica Acta: International Journal of Optics*, **31**(4), 403–413.

Stuckelberger, M., West, B., Nietzold, T., Lai, B., Maser, J. M., Rose, V. & Bertoni, M. I. (2017). *Journal of Materials Research*, **32**(10).

Yan, H., Conley, R., Bouet, N. & Chu, Y. S. (2014). *Journal of Physics D: Applied Physics*, **47**(26), 263001.

Yan, H., Kang, H. C., Conley, R., Liu, C., Macrander, A. T., Stephenson, G. B. & Maser, J. (2010). *X-ray Optics and Instrumentation*, **2010**.

Zozulya, A., Bondarenko, S., Schavkan, A., Westermeier, F., Grübel, G. & Sprung, M. (2012). *Optics express*, **20**(17), 18967–18976.
